# Polybasic Trafficking Signal Mediates Golgi Export, ER Retention or ER Export and Retrieval Based on Membrane-Proximity

**DOI:** 10.1371/journal.pone.0094194

**Published:** 2014-04-08

**Authors:** Hirendrasinh B. Parmar, Chris Barry, Roy Duncan

**Affiliations:** 1 Department of Microbiology & Immunology, Dalhousie University, Halifax, Nova Scotia, Canada; 2 Department of Biochemistry & Molecular Biology, Dalhousie University, Halifax, Nova Scotia, Canada; 3 Department of Pediatrics, Dalhousie University, Halifax, Nova Scotia, Canada; University of Missouri, United States of America

## Abstract

Trafficking of integral membrane proteins between the ER and Golgi complex, and protein sorting and trafficking between the TGN and endosomal/lysosomal compartments or plasma membranes, are dependent on *cis*-acting, linear amino acid sorting signals. Numerous sorting signals of this type have been identified in the cytoplasmic domains of membrane proteins, several of which rely on basic residues. A novel Golgi export signal that relies on a membrane-proximal polybasic motif (PBM) was recently identified in the reptilian reovirus p14 protein, a representative of an unusual group of bitopic fusion-associated small transmembrane (FAST) proteins encoded by fusogenic orthoreoviruses and responsible for cell-cell fusion and syncytium formation. Using immunofluorescence microscopy, cell surface immunofluorescence, and endoglycosidase H assays, we now show the p14 PBM can mediate several distinct trafficking functions depending on its proximity to the transmembrane domain (TMD). When present within 4-residues of the TMD it serves as a Golgi export signal, but when located at the C-terminus of the 68-residue p14 cytoplasmic endodomain it functions as an ER retention signal. The PBM has no effect on protein trafficking when located at an internal position in the cytoplasmic domain. When present in both membrane-proximal and -distal locations, the PBMs promote export to, and efficient retrieval from, the Golgi complex. Interestingly, the conflicting trafficking signals provided by two PBMs induces extensive ER tubulation and segregation of ER components. These studies highlight how a single trafficking signal in a simple transmembrane protein can have remarkably diverse, position-dependent effects on protein trafficking and ER morphogenesis.

## Introduction

Approximately one third of the human proteome comprises membrane proteins, which must be sorted and trafficked to the correct membrane compartment [1], [2]. Aberrant membrane protein trafficking is associated with several disease states [3]. Sorting and trafficking of integral membrane proteins is regulated by *cis*-acting sorting signals present in the protein cargo and *trans*-acting factors involved in vesicular transport. While numerous sorting signals, adaptor proteins and vesicle coat components involved in membrane protein sorting and trafficking have been identified [4], [5], our understanding of the mechanisms underlying membrane protein sorting is incomplete [6].

Integral membrane proteins generally begin their journey by co-translational insertion into the endoplasmic reticulum (ER), followed by transport in COPII-coated vesicles through the ER-Golgi intermediate compartment (ERGIC) to the Golgi complex. Numerous sorting signals, comprising short, linear amino acid sequences present in membrane protein cytoplasmic tails, regulate this anterograde transport [7]. ER export signals include di-basic, tri-basic, di-acidic, di-leucine and tyrosine-based signals, several of which interact with components of the COPII complex [8]–[12]. Short, degenerate tyrosine- and di-leucine sequence motifs also mediate trafficking from the *trans*-Golgi network (TGN), the main sorting hub for proteins destined to lysosomes, endosomes or the plasma membrane [13]. Similar to anterograde transport, linear sorting signals also mediate retention or retrieval of membrane proteins to the ER. Steady state accumulation of proteins in the ER can be achieved via ER retention signals, or by signals that interact with COPI for retrieval from the ERGIC or Golgi complex via retrograde flow [14].

The orthoreovirus fusion-associated small transmembrane (FAST) proteins are the smallest known membrane fusion proteins [15]. These nonstructural viral proteins evolved specifically to promote dissemination of the infection by inducing cell-cell fusion between virus-infected cells and neighboring uninfected cells [16], [17]. The FAST proteins are bitopic, integral membrane proteins whose single transmembrane domain (TMD) is flanked by small N-terminal ectodomains and equal-sized or larger C-terminal cytoplasmic endodomains [18]–[23]. In the absence of a cleavable signal peptide, the FAST protein TMD functions as a reverse signal anchor to direct insertion in ER membranes [24], followed by transit through the Golgi complex to the plasma membrane. We recently determined a polybasic motif (PBM) located four residues downstream of the p14 FAST protein TMD functions as a novel Golgi export signal [25]. Alanine substitution of the PBM (p14PA construct) leads to p14 accumulation in the Golgi complex and TGN, and mutagenic analysis revealed efficient Golgi export requires a minimum of three sequence-independent basic residues. Furthermore, introduction of the tri-basic motif into a Golgi-localized, chimeric ERGIC-53 protein directed export from the Golgi complex to the plasma membrane [25]. The p14 PBM is the first example of an autonomous, tri-basic signal required for Golgi export to the plasma membrane.

**Figure 1:**
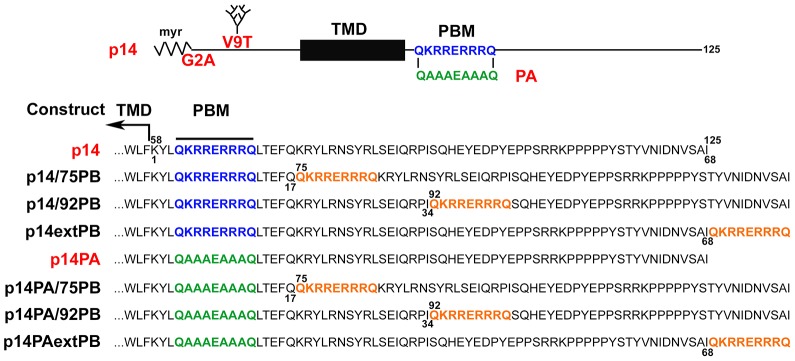
Motif arrangements in p14 and mutated p14 constructs used in this study. The top panel depicts motif arrangements in the full-length, 125-residue p14 protein, including N-terminal myristoylation (myr), transmembrane domain (TMD) and polybasic motif (PBM). The sequence of the PBM and the polyalanine substitution (PA) of this motif are shown. G2A and V9T are the locations of pint substitutions that eliminate the myristoylation motif (G2A) or introduce an N-linked glycosylation site (V9T), depicted as a branched tree. These p14 backbones (authentic p14, p14PA, p14-G2A, and p14-V9T) were used as templates for insertion of PBMs in various locations. The lower panel depicts the sequence of the p14 endodomain. Numbers on the top of the sequence indicate amino-acid positions relative to full-length protein. Numbers below the sequence indicate amino-acid position relative to the first residue in the endodomain. The boundary of the TMD and location of the polybasic motif (PBM) are indicated. The PBM was inserted in various locations in the endodomain (orange sequences), either in an authentic p14 backbone containing the membrane-proximal PBM (blue sequences), or in a p14PA backbone containing polyalanine substitution of the PBM (green sequences). The p14 and p14PA backbones contained either the G2A or V9T substitutions depicted above, as specified in the text and figure legends.

The p14 FAST protein is a small (125 residues), non-glycoslyated, single-pass, integral membrane protein (Fig. 1) that provides a simple system to explore pathways regulating plasma membrane trafficking. Preliminary analysis revealed replacement of the membrane-proximal PBM with a C-terminal version of this motif resulted in p14 accumulation in the ER [25], suggesting membrane-proximity may be a determining factor in the function of this novel sorting signal. To further explore this issue, the PBM was inserted at various positions within the 68-residue p14 endodomain (Fig. 1). Trafficking of these constructs was assessed using immunofluorescence microscopy, cell surface immunofluorescence, and glycosylation assays. Results indicate proximity of the PBM relative to the TMD and C-terminus dictates whether this sorting signal functions as a Golgi export signal, ER retention signal, or ER retrieval signal. Interestingly, when present in both membrane-proximal and –distal locations, the PBM induces extensive ER tubulation and alters distribution of a luminal KDEL ER marker.

## Results

### Polybasic Motif Functions as a Golgi Export Signal or ER Retention Signal Depending on Membrane-Proximity

**Figure 2:**
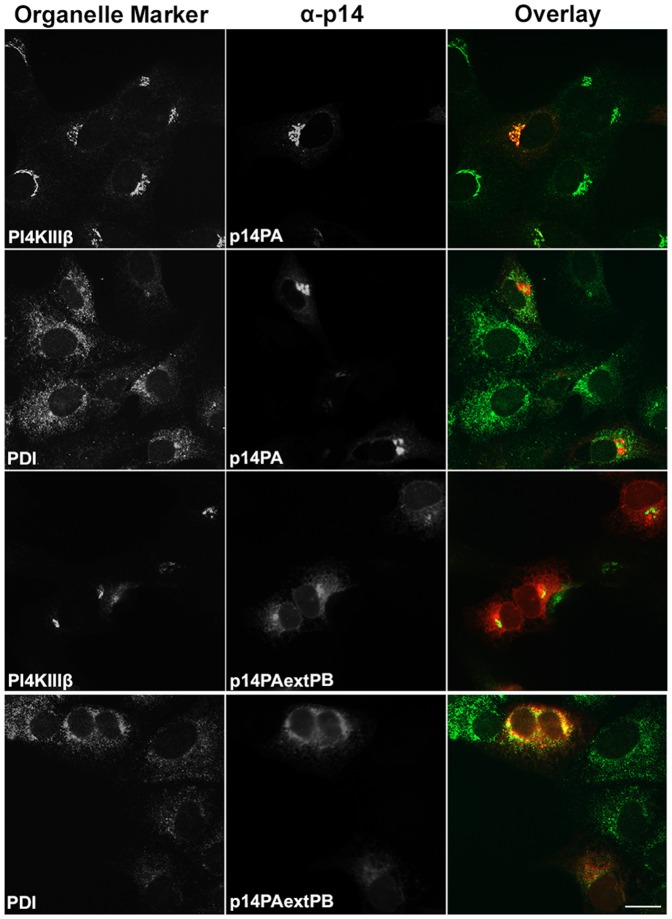
p14 accumulates in the Golgi complex or ER depending on PBM membrane-proximity. Vero cells transfected with p14PB or p14PBextPB in a p14-G2A backbone (see Fig. 1) were fixed and stained at 24 h post-transfection with anti-p14 antiserum (red) and the indicated organelle markers (green) for the Golgi (PI4KIIIβ) or the ER (PDI). Right column shows merged images. Scale bar  =  20 μm.

Recent analysis of p14PAextPB, a p14 construct containing alanine substitutions of the membrane-proximal PBM and an addition of the 9-residue PBM (QKRRERRRQ) to the C-terminus (Fig. 1), indicated this construct accumulated in the ER [25]. These studies were conducted using a p14-V9T backbone; the V9T substitution creates an N-linked glyscosylation signal that can be used to assess ER-Golgi trafficking of p14 by examining resistance to endoglycosidase H (endo H). Since glycosylation can contribute to membrane protein sorting and trafficking [26], we first sought to confirm this ER retention phenotype in a p14 backbone not subject to glycosylation. The same construct was therefore created in a p14-G2A background (Fig. 1); p14-G2A is a non-glycosylated, fusion-defective p14 construct that displays normal plasma membrane trafficking [21]. Immunofluorescence microscopy of Vero cells transfected with p14PAextPB-G2A resulted in intense colocalization with the ER marker protein disulfide isomerase (PDI), and limited colocalization with phosphatidylinositol 4 kinase IIIβ (PI4KIIIβ), a marker for the Golgi complex (Fig. 2). In contrast, p14PA-G2A (i.e., the same construct but lacking the appended C-terminal PBM) intensely colocalized with the Golgi marker PI4KIIIβ but showed limited colocalization with the ER marker PDI (Fig. 2). These results confirmed the PBM motif functions as a Golgi export signal when membrane-proximal, but not when membrane-distal, and that when present membrane-distal leads to p14 accumulation in the ER.

### Membrane Proximity Dictates Whether the PBM Functions as a Sorting Signal

**Figure 3:**
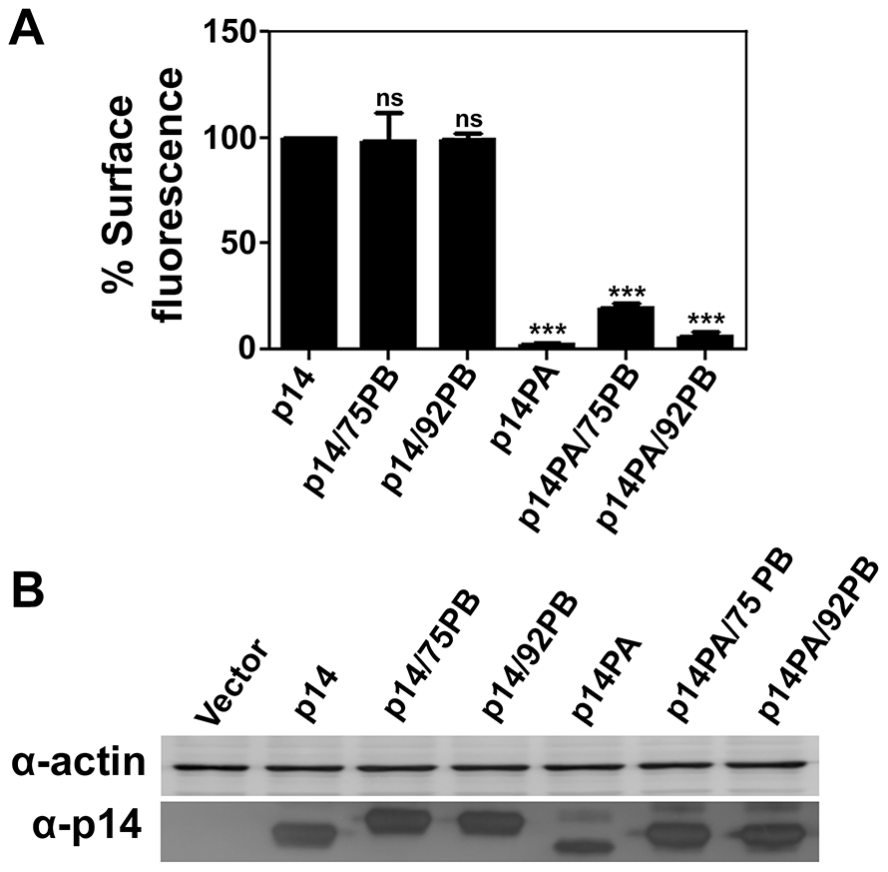
An internal PBM does not alter p14 trafficking to the plasma membrane. (A) QM5 cells transfected with p14-G2A or the indicated p14 mutants in a p14-G2A backbone (see Fig. 1) were surface stained at 24 h post-transfection using anti-p14ecto antiserum and Alexa-647 secondary antibody, and analyzed by flow cytometry. Percent cell surface fluorescence relative to p14 are presented as mean ± SEM from three independent experiments in triplicate. Statistical significance by one-way ANNOVA and Tukey post-test is shown relative to p14 (***p<0.005, ns-not significant). (B) QM5 cells transfected with p14 and the same constructs as in panel A were harvested at 8 h post-transfection and lysates were processed for western blotting using anti-p14 antiserum or anti-actin antibody.

**Figure 4:**
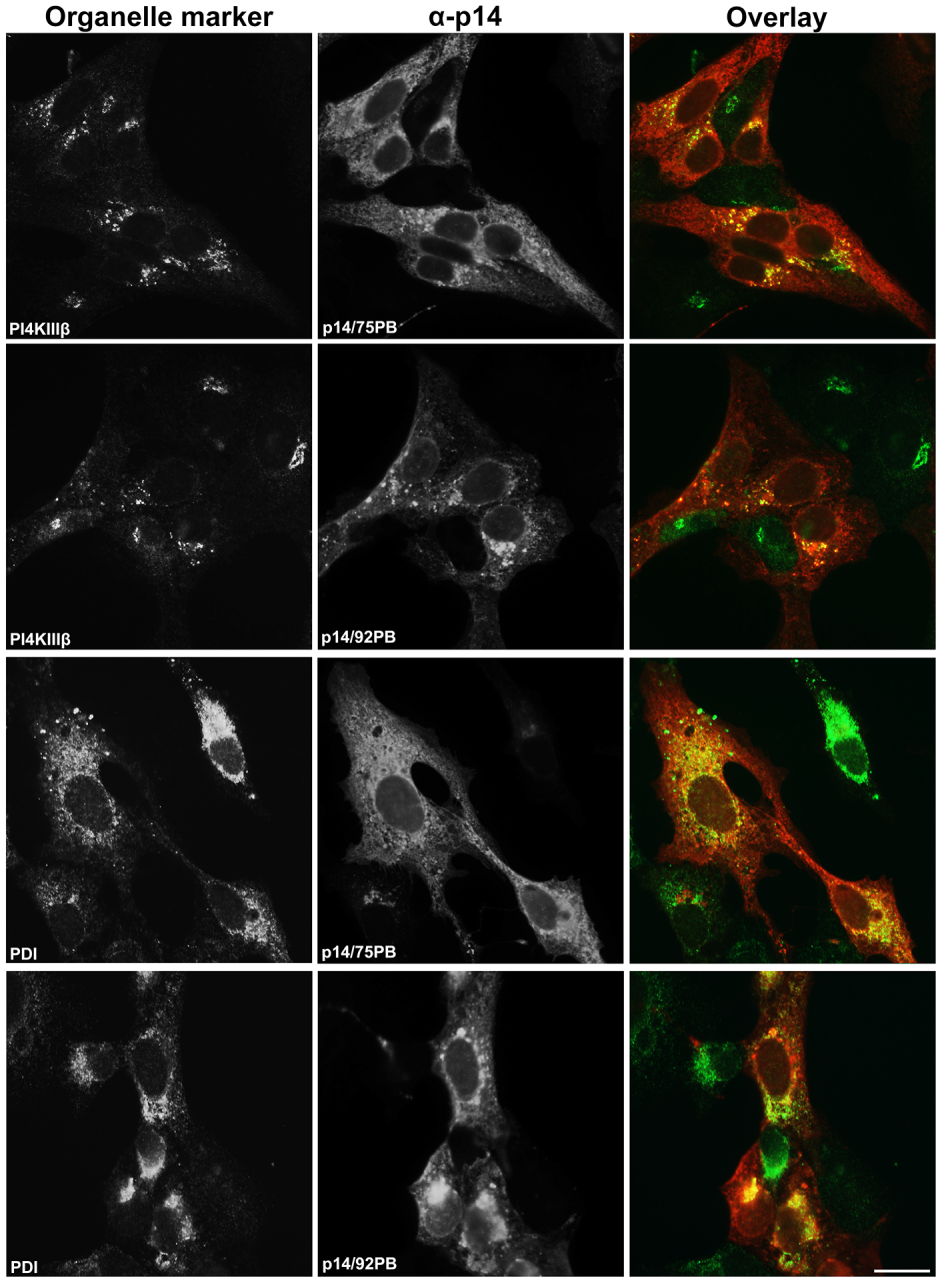
An internal PBM does not interfere with Golgi export function of a membrane-proximal PBM. Vero cells transfected with p14/75PB or p14/92PB in a p14-G2A backbone (see Fig. 1) were immunostained with anti-p14 antiserum (red) and the indicated organelle markers (green) as in Figure 2. Right column shows merged images. Scale bar  =  20 μm.

**Figure 5:**
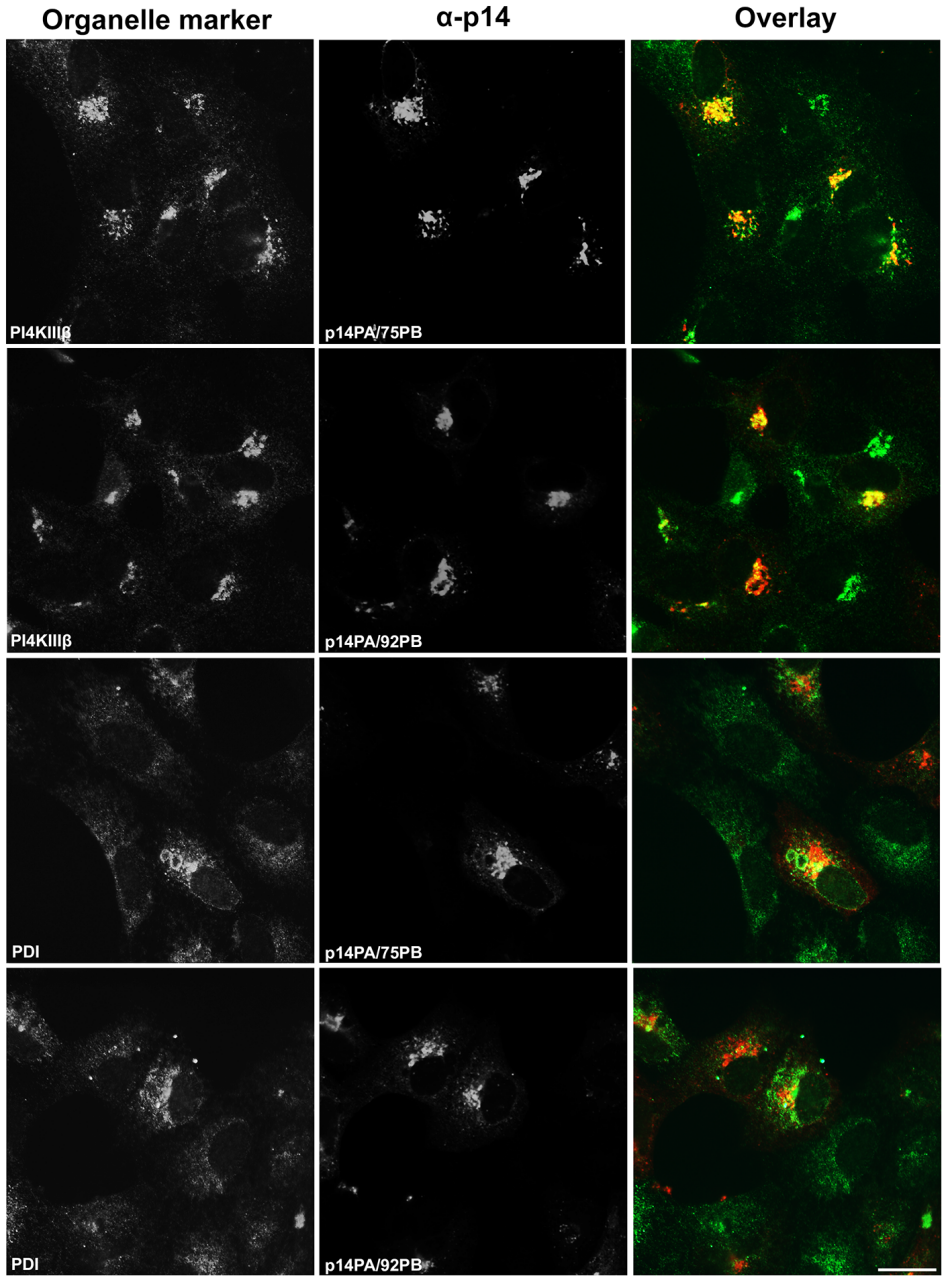
An internal PBM cannot function as a Golgi export signal. Vero cells transfected with p14PA/75PB or p14/PA92PB in a p14-G2A backbone (see Fig. 1) were immunostained as in Figure 2 using anti-p14 antiserum (red) and the indicated organelle markers (green). Right column shows merged images. Scale bar  =  20 μm.

To further examine membrane-proximity effects on the p14 PBM sorting signal, this motif was introduced at internal locations in the p14-G2A and p14PA-G2A endodomains (Fig. 1). Plasma membrane localization of these p14 constructs was quantified by flow cytometry using cell surface immunostaining with anti-p14ecto antiserum (recognizes the N-terminal p14 ectodomain). The PBM, when present 17 (p14PA/75PB-G2A) or 34 (p14PA/92PB-G2A) residues downstream of the TMD (corresponding to 51 or 34 residues upstream of the C-terminus, respectively), failed to promote p14 transport to the plasma membrane (Fig. 3A). The same insertions when present in p14-G2A (constructs p14/75PB-G2A and p14/92PB-G2A in Fig. 1) had no effect on p14 plasma membrane localization (Fig 3A). All constructs were expressed at similar steady state levels in cells, as indicated by western blotting (Fig. 3B). When examined by intracellular immunofluorescence microscopy, p14/75PB-G2A and p14/92PB-G2A both displayed diffuse cytoplasmic staining that radiated out to the cell periphery and partially colocalized with PI4KIIIβ and PDI (Fig 4). This staining pattern is typical of authentic p14 trafficking through the ER-Golgi pathway to the plasma membrane [25]. In contrast, p14PA/75PB-G2A and p14PA/92PB-G2A displayed partial co-localization with the PDI ER marker but intense co-localization with the PI4KIIIβ Golgi marker (Fig. 5), as did p14PA-G2A (Fig. 2). Thus, an internal PBM by itself cannot function to promote p14 Golgi export, nor does it interfere with the Golgi export function of a membrane-proximal PBM.

### Membrane-distal PBM Functions as ER Retrieval Signal that Dominates over Membrane-proximal PBM Golgi Export Signal

**Figure 6:**
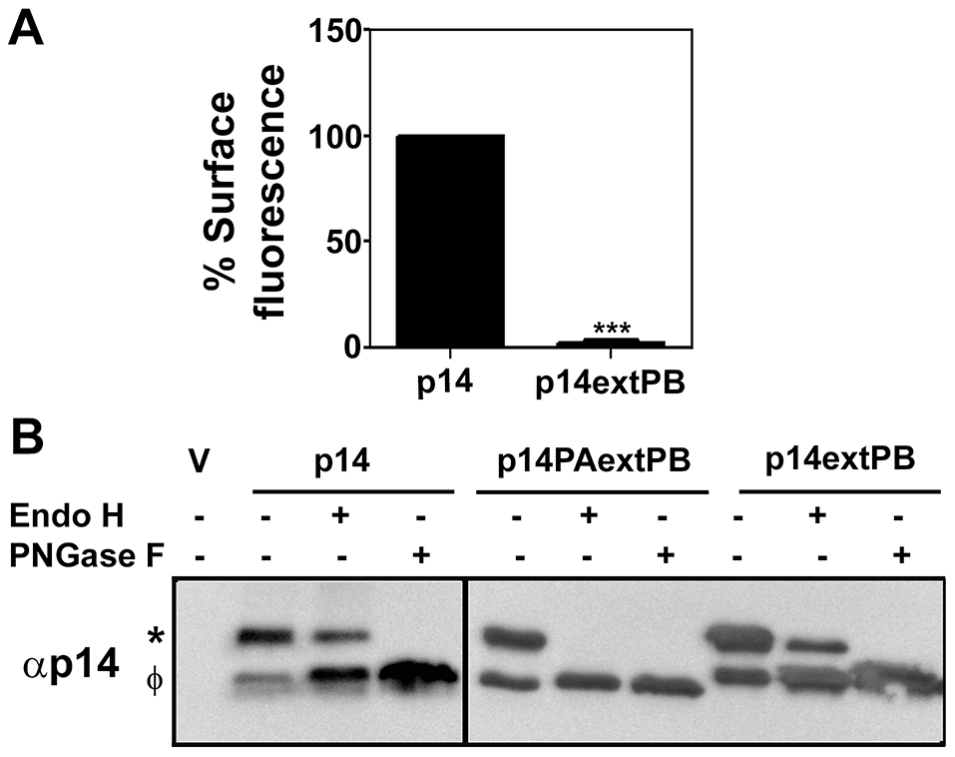
Membrane-distal and -proximal PBMs alter ER-Golgi p14 trafficking. (A) QM5 cells transfected with p14-V9T or p14extPB in a p14-V9T backbone (see Fig. 1) were surface stained at 24 h post-transfection using anti-p14ecto antiserum and Alexa-647 secondary antibody, and analyzed by flow cytometry. Percent cell surface fluorescence relative to p14-V9T is presented as mean ± SEM from three independent experiments in triplicate. Statistical significance from student t-test is shown relative to p14 (***p<0.005) (B) Cell lysates of QM5 cells transfected with empty vector (V), p14, p14PAextPB or p14extPB, all in a p14-V9T backbone, were left untreated or treated with endo H or PNGaseF and processed for western blotting with anti p14 antiserum. Glycosylated (*) and non-glycosylated (φ) p14 are indicated on left.

**Figure 7:**
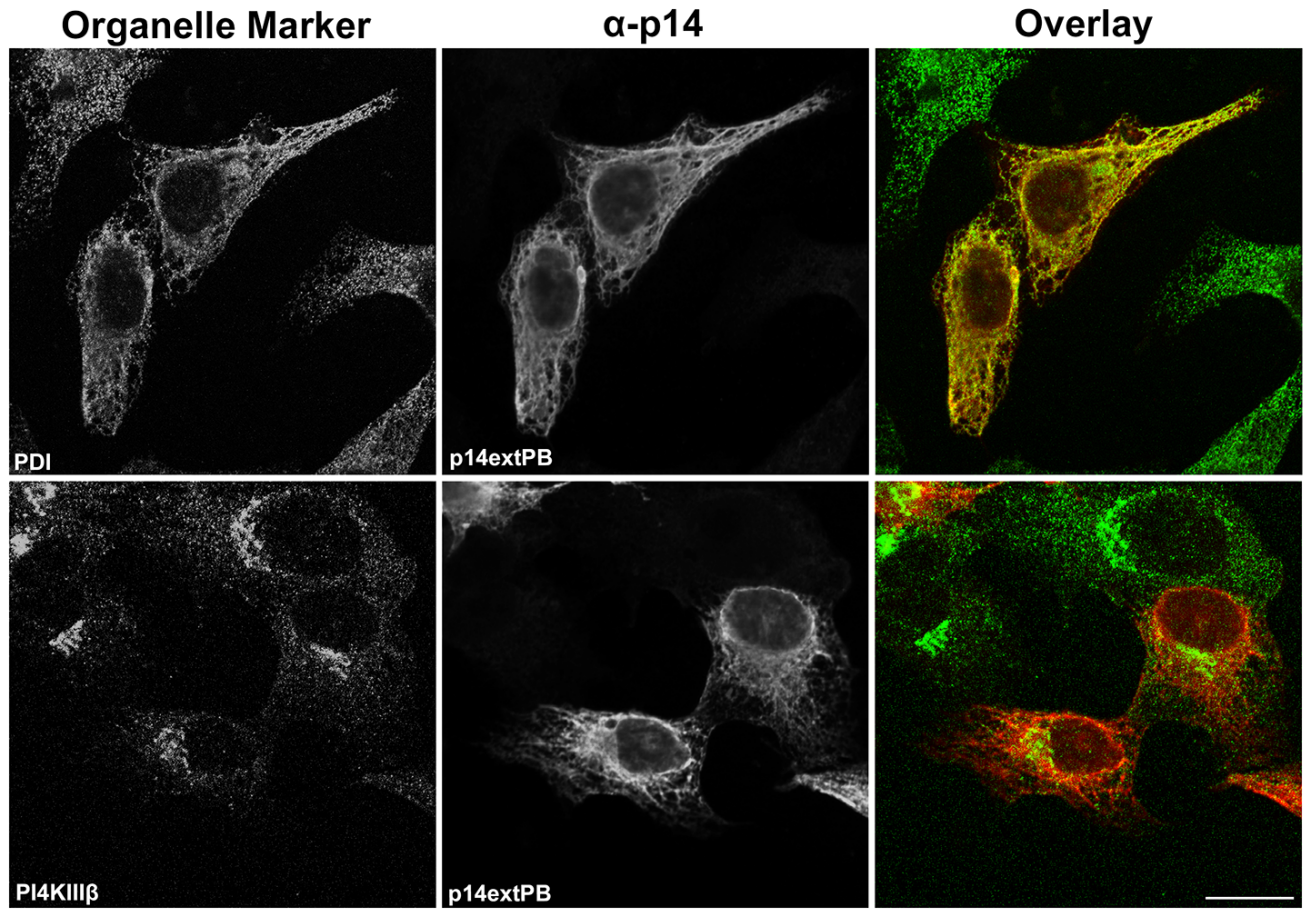
Membrane-distal PBM dominates over membrane-proximal PBM. Vero cells were transfected with p14extPBin a p14-V9T backbone and immunostained with anti-p14 antiserum (red) and the indicated organelle markers (green) as in Figure 2. Right column shows merged images. Scale Bar  =  20 μm.

Results indicated a membrane-proximal PBM functions as a Golgi export signal, while a C-terminal PBM leads to p14 accumulation in the ER (Fig. 2). To determine how p14 trafficking is affected by the presence of both membrane-distal and -proximal PBMs, the PBM was added to the C-terminus of p14-V9T (Fig 1). This p14extPB-V9T mutant was undetectable on the cell surface when analyzed by flow cytometry (Fig. 6A). Endo H assays were used to assess trafficking of this construct between ER and Golgi complex compartments, using p14-V9T and p14PAextPB-V9T constructs for comparison. Western blots detected two p14-V9T polypeptides (Fig. 6B); the slower migrating species was glycosylated, as indicated by sensitivity to PNGase F treatment, an amidase that cleaves both high-mannose and complex N-linked carbohydrates from glycoproteins. The slower migrating species was also partially resistant to endo H treatment, indicating transit to the Golgi complex and processing into an endo H-resistant complex oligosaccharide. As recently reported [25], p14PAextPB-V9T was similarly glycosylated but not processed to an endo H-resistant form (Fig. 6B), consistent with the microscopy results showing accumulation of this construct in the ER (Fig. 2). A C-terminal PBM in the absence of a membrane-proximal PBM therefore functions as an ER retention signal. In contrast, p14extPB-V9T generated an endo H-resistant form, similar to parental p14-V9T (Fig. 6B). This observation was apparent in two independent experiments, and indicated p14extPB-V9T is trafficked to the Golgi complex. When examined by immunofluorescence microscopy, p14extPB-V9T displayed nearly complete co-localization with the PDI ER marker and no obvious co-localization with the Golgi marker (Fig 7). Together, the endo H and microscopy results indicate a C-terminal p14 PBM can also function as an ER retrieval signal.

### Conflicting ER Retrieval and Golgi Export Signals Induce Extensive ER Tubulation

**Figure 8:**
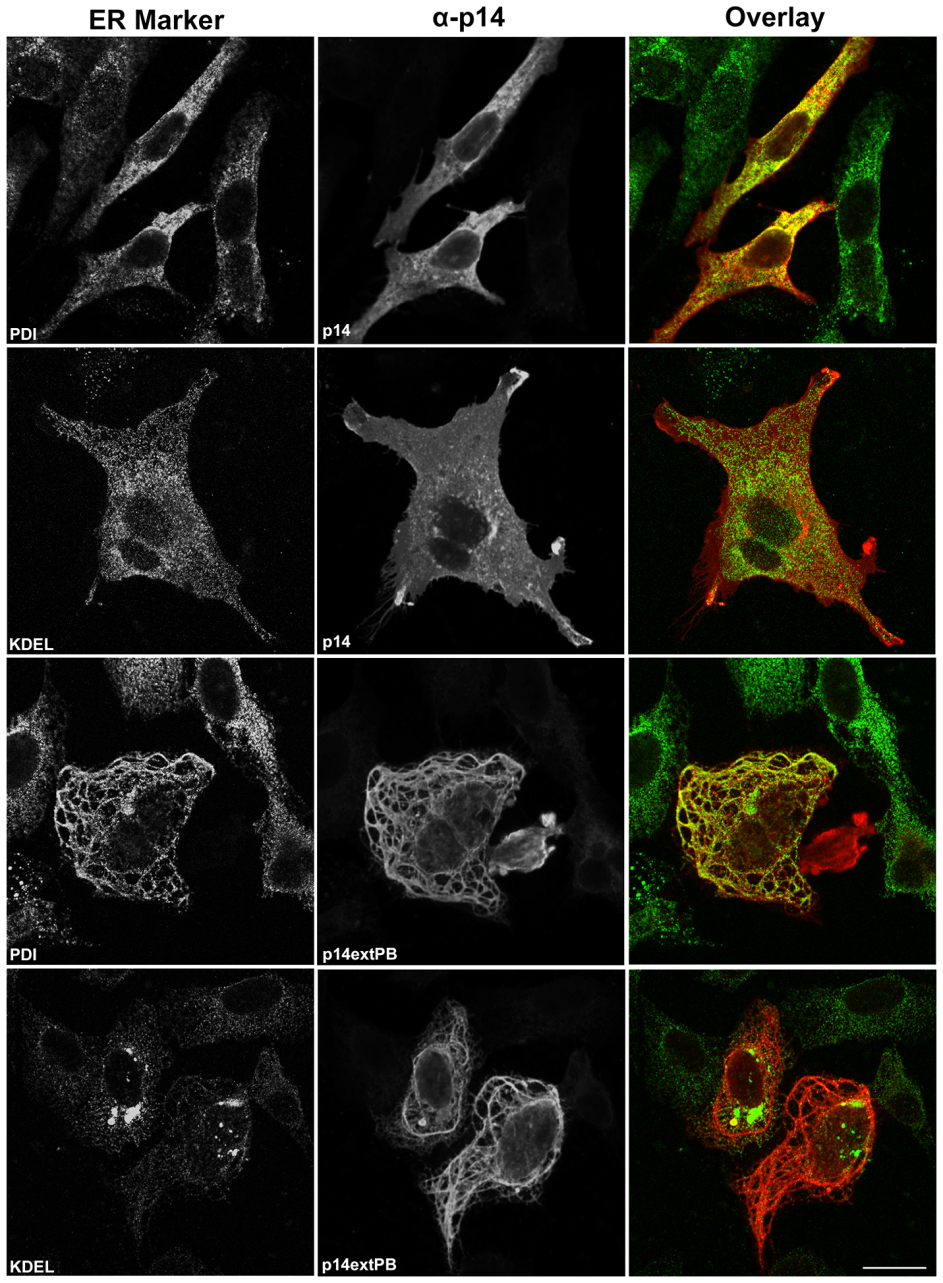
p14extPB induces ER tubulation and segregation. Vero cells transfected with p14-V9T or p14extPB in a p14-V9T backbone were immunostained with anti-p14 antiserum (red) and the indicated organelle markers (green) as in Figure 2. Right column shows merged images. Scale bar  =  20 μm.

Interestingly, the ER staining pattern of cells expressing p14extPB-V9T took on a decidedly tubular appearance, quite distinct from the punctate ER staining pattern in non-transfected cells evident in the same field of view (Fig. 7); appearance of the Golgi complex in these two cell types were indistinguishable. To more closely examine this ER phenotype, transfected cells expressing p14-V9T or p14extPB-V9T were immunostained for p14 and two different ER markers, PDI and KDEL. As shown in Fig 8, PDI and KDEL both displayed a punctate staining pattern throughout the cytoplasm in cells expressing p14-V9T. However, while the p14 staining pattern overlapped considerably with that of PDI, there was little evidence of detectable overlap in the p14 and KDEL staining patterns (Fig. 8). In marked contrast, PDI staining revealed an extensive web of tubulated ER in cells expressing p14extPB-V9T, and p14 perfectly colocalized with this reticular network. KDEL staining was also dramatically different from cells expressing p14-V9T, and was concentrated in a limited number of perinuclear aggregates that showed limited co-localization with p14extPB-V9T (Fig. 8). Thus, conflicting trafficking signals in a single transmembrane protein can induce dramatic changes in ER structure.

## Discussion

Cytoplasmic tails of numerous different membrane proteins contain sorting signals defined by basic residues, and these signals can direct ER export, retention or retrieval [27]–[30]. The p14 PBM is a newly discovered and rare example of a basic sorting signal mediating export from the Golgi complex, and preliminary analysis suggested this role is affected by membrane-proximity [25]. A more detailed analysis of positional bias revealed the p14 PBM can promote Golgi export and ER export, retention and retrieval based on its location within the 68-residue p14 cytoplasmic tail, and whether the PBM is present in one or two copies. Furthermore, when present in both membrane-proximal and membrane-distal locations, conflicting PBM-mediated trafficking signals dramatically alter ER morphology.

**Figure 9:**
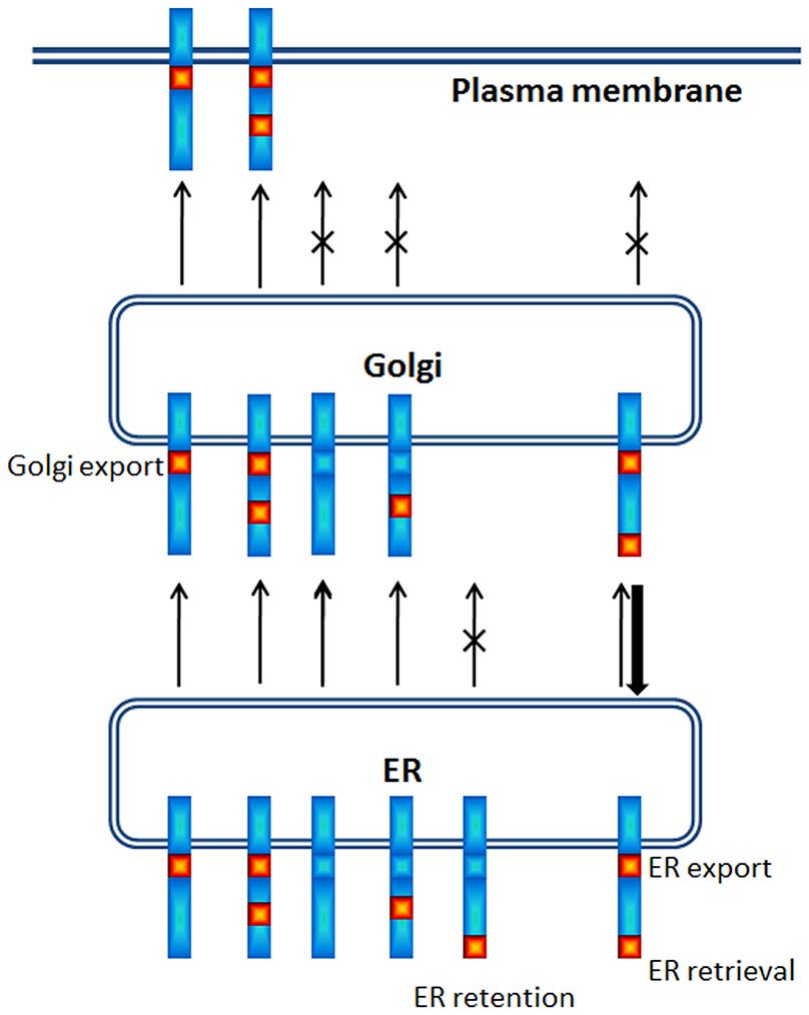
Summary of position-dependent effects of the PBM on p14 trafficking. Trafficking of p14 constructs (blue) containing the PBM (red) in membrane-proximal, C-terminal or internal locations in the p14 cytoplasmic endodomain, as described in the text, is depicted. Arrows indicate trafficking of the various constructs between the ER, Golgi and plasma membrane, with an X indicating inhibition and thin and thick arrows indicating relative strength of directional trafficking. The positional-dependent functions of the PBM at specific locations in Golgi export, ER retention, ER export or ER retrieval are indicated.

The present study revealed the p14 PBM can exert remarkably diverse, position-dependent effects on p14 trafficking (Fig. 9). As recently shown and confirmed here [25], p14 lacking a PBM fails to traffic to the plasma membrane and accumulates in the Golgi complex (Fig. 2). When present only at the C-terminus, the PBM inhibits ER exit (i.e., no acquisition of endo H-resistance by p14PAextPB) and leads to p14 accumulation in the ER (Figs. 2 and 6B). Thus, the p14 PBM functions as a Golgi export signal when membrane-proximal and as an ER retention signal when located C-terminal (Fig. 9), with the caveat that ER retention could also mean rapid retrieval from an ER-adjacent sorting compartment (e.g., the ERGIC). Unexpectedly, the Golgi export and ER retention activities of the PBM appeared to cancel out each other when present together. Endo H and surface expression assays indicated p14extPB is trafficked to the Golgi but not to the plasma membrane (Fig. 6), implying the C-terminal PBM did not promote ER retention and the membrane-proximal PBM did not mediate Golgi export. Instead, immunofluorescence microscopy indicated p14extPB accumulates in the ER (Fig. 7), suggesting the C-terminal PBM promotes ER retrieval of p14extPB and supersedes the Golgi export function of the membrane-proximal PBM. Concurrently, the membrane-proximal PBM in p14extPB appeared to assume a new role as an ER export signal. The PBM is not required for ER export, since p14PA efficiently exits the ER to the Golgi (Fig. 2), yet p14extPB is transported to the Golgi while p14PAextPB is retained in the ER, suggesting the membrane-proximal PBM in p14extPB promotes ER exit (Fig. 9). We previously noted a consistent decrease in the proportion of endo H-resistant p14PA relative to authentic p14, although the difference was not statistically significant [25]. It may be that a membrane-proximal PBM can enhance p14 exit from the ER at levels sufficient to at least partially override the C-terminal retention motif, thereby promoting p14extPB trafficking to the Golgi complex (Fig. 9).

Present results also indicate the PBM sorting signal is cryptic when positioned at internal locations in the p14 endodomain (Fig. 9). When located >17 residues from the TMD (p14PA/75PB) or >34 residues from the C-terminus or TMD (p14PA/92PB), the PBM had no effect on the Golgi export function of a membrane-proximal PBM (Fig. 4), and did not function as an ER retention signal in constructs lacking a membrane-proximal PBM (Fig. 5). The concept that basic sorting motifs are only active within a defined functional zone relative to the TMD and C-terminus of membrane proteins has been well established [31]. These positional effects on ER trafficking are attributable to interactions with different components of COPI and COPII complexes [9], [10], [32]. For example, KxKxx and KKxx motifs must be C-terminal to allow interactions with the α/β′/ε B-subcomplex of COPI [29], [32], [33], and these motifs mediate ER retention/retrieval when located 17 residues, but not 37 residues, distal from the TMD [31]. Conversely, φRxR motifs (φRxR; φ is an aromatic or bulky hydrophobic residue) involved in ER retention are not dependent on a C-terminal location, they interact with the Sar1 or Sec24 components of COPII complex, and mediate ER retention when located >45 residues from the C-terminus but not when positioned <25 residues from the TMD [28], [31], [34].

Our positional studies indicate the p14 PBM shares features with the well-characterized di-lysine (KKxx and KxKxx) and di-arginine (φRxR) ER retention/retrieval motifs, but is distinct from both. The p14 PBM mediates ER retention/retrieval when C-terminal, similar to di-lysine motifs. However, the p14 PBM (QKRRERRRQ) does not adhere to the strict requirement for lysines in positions -3 and -4 or -5 relative to the C-terminus [27], [29]. The di-lysine motifs also function best when the C-terminus is within ∼17–37 residues of the TMD, while the PBM is functional when located at the C-terminus of the 68-residue p14 endodomain. The ability of the p14 PBM to function as an ER retention/retrieval signal when membrane-distal is a feature shared with φRxR di-arginine motifs. However, an acidic residue in the φ or X positions usually renders the signal inactive [31]; the glutamic acid residue in the p14 PBM occurs in either of these positions in all possible φRxR combinations. The φRxR ER retention motif also functions when located at internal positions in the cytoplasmic tails of membrane proteins [28], which is not the case for the p14 PBM. We note that in addition to these two well-described ER retention/retrieval motifs, an unrelated tri-arginine motif is also involved in ER retrieval [35]. The p14 PBM may represent another example of a novel ER trafficking motif based on basic residues.

Lastly, we noted a dramatic reorganization of the ER in cells expressing p14 containing conflicting ER trafficking signals. The p14extPB construct induced extensive ER tubulation, and distribution of the KDEL marker was strikingly altered in p14extPB cells, with a broadly distributed, punctate staining pattern collapsing into a few large perinuclear patches (Figs. 7 and 8). This phenotype is remarkably similar to that recently reported for cells treated with the small molecule inhibitor dispergo [36]. Dispergo induces ER tubulation, loss of ER cisternae, and generation of ER patches. These patches contain large amounts of condensed ER tubules, the Sec61β component of the ER translocon, and the lumenal ER markers KDEL and calreticulin. Unlike dispergo, which inhibits ER export leading to loss of the Golgi complex, ER export was still functional in cells expressing p14extPB (p14extPB-V9T is modified to an endo H-resistant form in the Golgi complex; Fig. 6B) and the Golgi complex remained intact (Fig. 7). The basis for the ER phenotype induced by dispergo or p14extPB is unknown. One possibility is that p14extPB activates reticulons, which interact with DP1/Yop1p to induce or stabilize membrane curvature leading to ER tubulation [37]–[39]. Reticulon overexpression results in ER tubulation and segregation of ER markers, including displacement of the lumenal KDEL marker, similar to what occurs in cells expressing p14extPB. Another interesting possibility involves inhibiting homotypic ER fusion. Formation and maintenance of the ER involves fusion of the tip of one tubule to the side of another, creating three-way junctions and an irregular polygonal ER network [40]. Atlastin, a dynamin-like GTPase that induces membrane curvature and fusion, is intimately involved in this fusion event [41], [42]. Since p14-induced cell-cell fusion and atlastin-mediated ER fusion require membrane curvature in the opposite directions, accumulation of p14extPB in the ER might inhibit atlastin-mediated ER fusion leading to a decrease in the polygonal ER arrangement and accumulation of long ER tubules. Additional studies aimed at discerning the mechanism by which p14extPB alters ER morphology and the molecular basis for the diverse position-dependent effects of the p14 PBM on membrane protein trafficking seem likely to provide additional insights into these essential cellular processes.

## Materials and Methods

### Cells, Clones and Antibodies

Vero cells used for microscopy and QM5 cells used for western blotting and cell surface fluorescence were maintained in medium 199 with Earle's salts supplemented with 5 or 10% fetal bovine serum (FBS), respectively. Vero cells were obtained from the American Type Culture Collection (ATCC). QM5 cells were obtained from Charles Ordahl [43]. Rabbit polyclonal anti-p14 and anti-14 ectodomain (p14ecto, residues 5–31) antisera were previously described [21], [44]. Antibodies against actin (Sigma), PI4KIIIβ (BD Transduction Laboratories), KDEL (Stressgen) and PDI (Stressgen), and horseradish peroxidase (HRP)-conjugated goat anti-rabbit (Jackson Immunoresearch), Alexa 488-conjugated goat anti-mouse and Alexa 647-conjugated goat anti-rabbit secondary antibodies (Life Technologies) were obtained from the indicated suppliers.

The p14 cDNA cloned into pcDNA3, and the non-fusogenic p14-V9T (point substitution introduces a functional glycosylation signal) and p14-G2A (point substitution eliminates the myristoylation consensus sequence) constructs were described previously [21], as were p14PA (alanine substitutions of the PBM) and p14PAextPB (p14PA with the PBM added to the C-terminus) in a p14-V9T backbone [25]. The Quick-Change (Stratagene) method was used according to manufacturer's specifications to introduce the G2A substitution into these constructs. The p14extPB was created using p14-V9T or p14-G2A as templates for PCR with a reverse primer that added the PBM to the C-terminus of the protein. The PBM (QKRRERRRQ) was inserted between p14 residues 74–75 (p14/75PB) or 91–92 (p14/92PB) in the p14-G2A or p14PA-G2A backbones using a reverse PCR primer containing the PBM nucleotide sequence.

### Western Blotting and Endoglyosidase H Assays

Multi-well plates containing subconfluent QM5 cells were transfected with plasmid DNA using Lipofectamine LTX (Life Technologies) according to manufacturer's instructions. For western blotting, cells were lysed at 8 h post-transfection with RIPA buffer (50 mM Tris pH 8.0, 150 mM NaCl, 1 mM EDTA, 1% NP-40, 0.5% IGEPAL) containing 1 μM protease inhibitors (aprotinin, pepstatin, and leupeptin) for 45 min on ice. Equivalent protein loads, as determined by Lowry assays (Biorad), were analyzed by SDS-PAGE (15% acrylamide) and western blotting using anti-p14 antiserum (1∶20,000) or anti-actin antibodies (1∶2,500) and horseradish peroxidase (HRP)-conjugated goat anti-rabbit secondary antibody (1∶10,000). For the endoglycosidase H (endo H) assays, cells were lysed at 24 h post-transfection, and prior to SDS-PAGE lysates were treated at 37°C for 2 h with endo H or N-glycosidase F (PNGase F) according to manufacturer's specifications (New England Biolabs). Membranes were developed using ECL-plus reagent (GE Healthcare) and imaged on a Typhoon 9410 variable mode imager (Amersham) or a Kodak 4000 mm Pro CCD imager.

### Cell Surface Immunofluorescence

At 24 h post-transfection, live QM5 cells were incubated at 4°C for 30 min in blocking buffer (5% normal goat serum, 1% bovine serum albumin (BSA), 0.02% NaN_3_ in HBSS) and then immunostained for 1 h at 4°C with a 1∶1000 dilution of anti-p14ecto antiserum and a 1∶2,000 dilution of Alexa 647-conjugated goat anti-rabbit secondary antibody. Cells were resuspended in phosphate buffered saline (PBS) containing 10 mM EDTA, fixed with 3.7% formaldehyde, and 10,000 cells were analyzed by flow cytometry (Becton Dickinson FACSCalibur) using De-Novo software. A fluorescence gate was set such that <5% of cells transfected with empty vector (negative control) were positive, the same gate was applied to quantify surface fluorescence of p14-transfected cells, and background fluorescence from vector-transfected cells was subtracted before calculating mean percent surface fluorescence.

### Intracellular Immunofluorescence

Vero cells cultured on glass coverslips (Fisher) were transfected with jetPRIME (PolyPlus Transfection) according to the manufacturer's instructions, and at 24 h post-transfection cells were fixed in 3.7% formaldehyde for 20 min at room temperature, permeabilized for 20 min at room temperature with 0.1% Triton X-100, blocked for 30 min in blocking buffer, stained with rabbit anti-p14 antiserum (1∶200) and with 1∶1,000 dilutions of mouse monoclonal antibodies against a Golgi marker (PI4KIIIβ) or ER markers (PDI or KDEL), and subsequently with 1∶1,000 dilutions of Alexa 488-conjugated goat anti-mouse and Alexa 647-conjugated goat anti-rabbit secondary antibodies. Coverslips were mounted on glass slides using fluorescence mounting medium (Dako) or Prolong gold antifade reagent (Molecular Probes), then visualized and photographed using a Zeiss LSM 510 META confocal microscope or a Zeiss Axioplan II MOT and AxioCam HRC Colour Camera. Images were acquired with either 63× or 100× magnification objectives.
